# The Video intervention to Inspire Treatment Adherence for Life (VITAL Start): protocol for a multisite randomized controlled trial of a brief video-based intervention to improve antiretroviral adherence and retention among HIV-infected pregnant women in Malawi

**DOI:** 10.1186/s13063-020-4131-8

**Published:** 2020-02-19

**Authors:** Maria H. Kim, Tapiwa A. Tembo, Alick Mazenga, Xiaoying Yu, Landon Myer, Rachael Sabelli, Robert Flick, Miriam Hartig, Elizabeth Wetzel, Katie Simon, Saeed Ahmed, Rose Nyirenda, Peter N. Kazembe, Mtisunge Mphande, Angella Mkandawire, Mike J. Chitani, Christine Markham, Andrea Ciaranello, Elaine J. Abrams

**Affiliations:** 1 Baylor College of Medicine International Pediatric AIDS Initiative, Texas Children’s Hospital, Houston, TX USA; 2 Baylor College of Medicine Children’s Foundation Malawi, Lilongwe, Malawi; 3 University of Texas Medical Branch at Galveston, Galveston, TX USA; 4 Division of Epidemiology and Biostatistics, School of Public Health and Family Medicine, University of Cape Town, Cape Town, South Africa; 5 HIV Unit, Malawi Ministry of Health, Lilongwe, Malawi; 6 Health Promotion & Behavioral Sciences, The University of Texas School of Public Health, Houston, TX USA; 7 Division of Infectious Diseases, Department of Medicine; Medical Practice Evaluation Center; both at Massachusetts General Hospital, Boston, MA USA; 8 ICAP at Columbia, Mailman School of Public Health and Vagelos College of Physicians and Surgeons, Columbia University, New York, NY USA

**Keywords:** HIV, ART (antiretroviral therapy), Retention, Adherence, PMTCT (prevention of mother-to-children transmission), Video

## Abstract

**Background:**

Improving maternal antiretroviral therapy (ART) retention and adherence is a critical challenge facing prevention of mother-to-child transmission (PMTCT) of HIV programs. There is an urgent need for evidence-based, cost-effective, and scalable interventions to improve maternal adherence and retention that can be feasibly implemented in overburdened health systems. Brief video-based interventions are a promising but underutilized approach to this crisis. We describe a trial protocol to evaluate the effectiveness and implementation of a standardized educational video-based intervention targeting HIV-infected pregnant women that seeks to optimize their ART retention and adherence by providing a VITAL Start (Video intervention to Inspire Treatment Adherence for Life) before committing to lifelong ART.

**Methods:**

This study is a multisite parallel group, randomized controlled trial assessing the effectiveness of a brief facility-based video intervention to optimize retention and adherence to ART among pregnant women living with HIV in Malawi. A total of 892 pregnant women living with HIV and not yet on ART will be randomized to standard-of-care pre-ART counseling or VITAL Start. The primary outcome is a composite of retention and adherence (viral load < 1000 copies/ml) 12 months after starting ART. Secondary outcomes include assessments of behavioral adherence (self-reported adherence, pharmacy refill, and tenofovir diphosphate concentration), psychosocial impact, and resource utilization. We will also examine the implementation of VITAL Start via surveys and qualitative interviews with patients, partners, and health care workers and conduct cost-effectiveness analyses.

**Discussion:**

This is a robust evaluation of an innovative facility-based video intervention for pregnant women living with HIV, with the potential to improve maternal and infant outcomes.

**Trial registration:**

ClinicalTrials.gov, NCT03654898. Registered on 31 August 2018.

## Background

In 2011, Malawi introduced Option B+ (B+), a policy of test-and-treat universal lifelong antiretroviral therapy (ART) for pregnant and breastfeeding women. After implementation, maternal ART uptake increased sevenfold, the proportion of pregnant women receiving ART increased from 49% in the pre-B+ era to 89% in 2016, and vertical transmission rates declined [1–6]. Recognizing these benefits, the World Health Organization endorsed B+, and the majority of high-prevalence countries are implementing B+. In part, due to the success of B+, countries have transitioned to universal treatment for all adults living with HIV infection.

Although the B+ policy resulted in significant gains in uptake of ART, suboptimal ART adherence and retention in care remain concerning. Only 59% of pregnant women initiated on ART were retained in care after 2 years, with the greatest losses occurring soon after initiating treatment [4, 7]. Of those retained, only two-thirds achieved adequate adherence [4, 8]. Drops in retention shortly after ART initiation have also been reported from other high-prevalence settings in the developing world [9–14].

Barriers to maternal retention and adherence are multifactorial. B+ was a radical paradigm shift, where individuals who felt healthy were told to start lifelong ART. While partner involvement can improve service uptake and adherence, women received little support in disclosing their status to partners [15, 16]. For the health system, rapid ART expansion occurred despite minimal increases in the health workforce. Overextended staff had to absorb a new flood of patients, resulting in poor pre-ART education, long wait times, increased health care worker (HCW) burnout, and frustrating provider-patient interactions [8, 17–20].

Several interventions to improve retention and adherence among HIV-positive pregnant and breastfeeding women have been studied [21–41]. The most effective examples are supporter interventions (by peer supporters or community health workers) and the use of short message service (SMS) technology, but these approaches require dedicated effort for only modest and temporary gains [21–32, 35, 42–46]. Other modalities include integration of prevention of mother-to-child transmission (PMTCT) services with postnatal care [47, 48], which helps to address retention further along the cascade. There is a lack of evidence describing interventions that specifically target the first encounter, which is critical in preventing the very early disengagement characteristic of B+.

There is an urgent need for interventions to improve retention and adherence that can be feasibly implemented in overburdened health systems. Brief video-based interventions are a promising but underutilized approach to this crisis. They have a strong track record in improving health knowledge, supporting partner disclosure, increasing treatment adherence, and fostering behavior change among low literacy patients [49–55]. Video interventions are scalable due to limited costs after creation [56]. Messages can be woven into engaging storylines, piloted to ensure cultural relevance, and delivered at critical teachable moments [53]. Videos can be provided in high-volume clinics to deliver standardized messages while freeing up HCW time. While video-based interventions have potential to address barriers to ART retention and adherence, they have seen limited use in Africa’s HIV pandemic.

In recognizing the critical need for evidence-based, cost-effective, and scalable interventions to improve maternal adherence and retention, our study team developed a brief video-based intervention that provides pregnant women living with HIV a VITAL Start (Video intervention to Inspire Treatment Adherence for Life) at the critical moment before committing to lifelong ART. The pilot demonstrated that the VITAL Start was very feasible to implement, highly accepted by both pregnant women and HCWs, and resulted in excellent knowledge acquisition and better short-term self-reported adherence as compared to the control [57]. In the present trial, we aim to rigorously evaluate the effect of VITAL Start compared to standard of care (SOC) on the primary composite outcome of retention and adherence in a multisite randomized controlled trial. If effective, VITAL Start offers an innovative, scalable tool to address the current crisis of maternal retention and adherence while supporting the overburdened health system in Malawi and similar settings by liberating HCW time.

## Methods/design

**Fig. 1:**
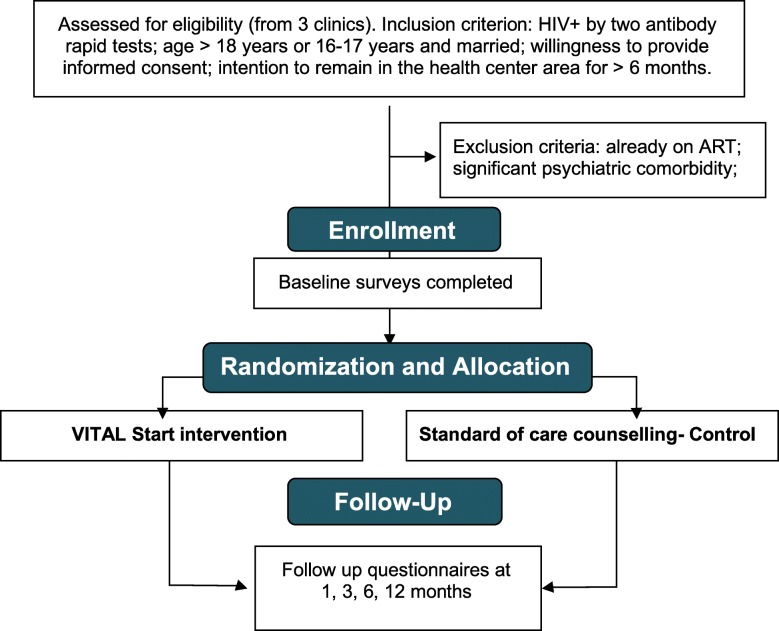
Study Flow Diagram, as per Consolidated Standards of Reporting Trials (CONSORT)

This randomized controlled trial will evaluate the effectiveness of the VITAL Start intervention on the primary composite outcome of retention and adherence at three sites in Malawi. These methods are based on protocol version 4.0 (updated on 23 August 2018). Figure 1, Study Flowchart.

### Trial setting

The trial will take place in Malawi, a landlocked country of 17 million people in sub-Saharan Africa. Malawi’s per capita gross national income is $340 USD, and it has an adult literacy rate of 62% [58, 59]. Almost all pregnant women (95%) attend an antenatal care (ANC) clinic at least once in pregnancy, and HIV status is determined in more than 90% of ANC attendees through a routine opt-out approach [3, 60]. Adult HIV prevalence in Malawi is approximately 11%, and combination ART is free for all patients with a confirmed diagnosis (defined as two serial positive results from rapid antibody-based tests on whole blood). The most commonly used first-line regimen for all patients (including pregnant women) is a once-daily fixed dose combination of efavirenz, lamivudine, and tenofovir disoproxil fumarate.

The trial will take place at three sites: two urban health facilities in the capital of Lilongwe (Area 25 and Kawale Health Centers) and one rural district hospital in the southern district of Mangochi (see Table 1). All sites are high-volume public Ministry of Health (MOH) facilities that were selected in consultation with the MOH.

**Table 1:** Trial site characteristics

Site	HIV prevalence at ANC (%)	HIV-positive women attending ANC per year, *n* (%)			Proportion retained after initiation (%)	
		*On ART*	*Not on ART*	*Total*	*6 months*	*12 months*
Kawale Health Center	12.4^a^	191 (38.9)	300 (61.1)	491	68^b^	57^b^
Area 25 Health Center	11.2	303 (51.9)	281 (48.1)	584	62	58
Mangochi District Hospital	12	288 (47.8)	315 (52.2)	603	46^c^	53^c^

All data from Malawi Ministry of Health HIV Unit quantitative data reports, calendar year 2015 unless otherwise noted due to missing data ^a^Included data from 2014 quarter 4 due to missing data in 2015 quarter 1 ^b^Included data from 2014 quarter 3 due to missing data in 2015 quarter 1 ^c^Using data from calendar year 2016

### Recruitment of participants

We will recruit 892 pregnant women living with HIV presenting to ANC clinics. Research assistants (RAs) assigned to each facility will deliver health talks to all patients attending ANC before the clinic begins, describing the trial and inviting interested patients to participate. RAs will also work with facility staff to identify eligible participants. To be eligible for trial participation, individuals must meet all of the following inclusion criteria:

1. Willing to provide informed consent
2. Currently pregnant, verified by palpation or urine pregnancy test if not palpable
3. HIV-positive, verified by two positive rapid antibody-based tests
4. Able to understand Chichewa
5. Age 18 or older. Individuals age 16–17 may participate only if they are either married or have a child
6. Not currently on ART. Individuals previously on ART but who disengaged from care prior to the index pregnancy are eligible
7. Plans to stay in the trial catchment area and receive ART and ANC services from the health facility of enrollment for at least 6 months.

Exclusion criteria include any of the following:

1. Already on ART
2. Pre-existing psychiatric comorbidity that would impair ability to provide informed consent.

Participants will be randomized in a 1:1 fashion to the SOC or VITAL Start arms. In terms of HIV/ART care received, the only difference between the arms will be the type of pre-ART counseling received.

### Standard-of-care pre-ART counseling

Participants randomized to SOC will receive the SOC pre-ART initiation education from a clinic health worker using the National ART Counseling Flip Chart [61], an educational tool with pictures on one side and key message prompts on the other. Topics include the basics of ART and the importance of adherence. Complete review of the flip chart typically takes one hour. To reduce contamination, VITAL Start and SOC will be delivered in separate areas.

### VITAL Start intervention pre-ART counseling

Participants in the VITAL Start arm will receive a 37-min intervention (27-min video and 10-min scripted RA-delivered individual counseling session). The video, titled Chiyembekezo (“Hope” in Chichewa), was developed through an iterative multistep process involving a team of persons living with HIV, researchers, clinicians, MOH staff, and other stakeholders. In conceptualizing the video, three behavior-determinant models were selected based on their demonstrated ability to promote behavior change in HIV/STD interventions:

1. Social cognitive theory, to address social influences
2. The theory of planned behavior, to address the cognitive aspects of how patients may conceptualize health threats and appraise barriers to or facilitators of lifelong retention and adherence to ART
3. The information-motivation-behavioral (IMB) skills model, which asserts that knowledge must be linked to both motivation and behavioral skills to overcome barriers critical to achieve adherence [57].

Development of video content was through a community participatory approach to ensure that the content was acceptable and engaging. Focus groups with people living with HIV/AIDS and local clinicians identified three key subject areas the video should target: (1) the importance of starting lifelong ART while feeling healthy; (2) management of ART side effects; (3) partner disclosure.

An advisory group of HCWs, ART providers, and MOH staff consulted at all stages of video development. Content was generated through a partnership with In Tune for Life, a nonprofit organization with expertise producing health promotion videos. The finished video depicts an urban Malawi setting with a female protagonist who is pregnant and newly diagnosed with HIV at ANC clinic. The film depicts her anxieties about her health, protecting her baby, disclosing to her husband, and remaining on ART for life. A nurse and close friend encourage her to disclose her status to her partner and remain adherent. Through their reassurances, the protagonist is empowered in her relationship with her husband and prepared to take ART for life.

Interventions for both arms (VITAL Start and SOC) occur upon trial enrollment at the ART initiation visit. Due to the one-off nature of the trial intervention, there are no criteria for discontinuing or modifying allocated interventions for a given trial participant after randomization. Participants will not be allowed to switch arms if they desire a different intervention after allocation. All other clinical care will be carried out according to MOH guidelines.

### Contamination and intervention fidelity

To help ensure treatment fidelity and reduce contamination, we have adopted practices recommended by the National Institutes of Health (NIH) Behavior Change Consortium Treatment Fidelity Workgroup in five areas: trial design, training, delivery of treatment, and receipt of and enactment of treatment skills [62]. Multiple strategies were developed, tested, and revised; key strategies are listed in Table 2. In brief, we will use two fidelity checklists to monitor, measure, and ensure fidelity to the VITAL Start intervention. The two checklists are an Observer Intervention Fidelity Checklist (completed by an RA observing the VITAL Start intervention) and a Self-Administered Intervention Fidelity Checklist (completed by the RA who is administering the intervention). An overall fidelity score will be determined to assess the level of fidelity maintained based upon four key measurement criteria as outlined in Dusenbury, et al. [65]: adherence, dose, quality of delivery, and participant responsiveness. Other fidelity measures are outlined in Table 2.

**Table 2:** Methods to reduce contamination during all phases of the proposed research and ensure intervention fidelity

Trial design and pre-implementation	
▪ Increased sample size to account for potential contamination (up to 15%)	
▪ Focus group discussions and literature reviews have explored types, extent of, and measures to reduce possible contamination sources. Overall risk of contamination deemed to be very small	
▪ Focus group discussions identified video recall items to measure contamination	
▪ Trial staff and trial clinic training emphasizes the implications of contamination, how to avoid it, how to address questions using non-biased explanations, and the need to ensure VITAL Start and SOC are delivered in separate places	
Implementation	
▪ VITAL Start and SOC occur in separate locations, *and intervention is delivered by trained RAs*	
▪ Participants will be escorted between counseling and survey locations	
▪ *Level and extent of patient-reported contamination is measured at follow-up visits*	
▪ Video is on password-protected tablet that is stored at the health facility and not available through other channels	
▪ Information packs with VITAL Start materials are clearly labeled and stored separately from SOC materials	
▪ Any accidental contamination by trial or health facility staff will be recorded on Unanticipated Problems Forms that are reviewed monthly at site-level meetings and supervisions	
▪ Site Research Supervisor to confirm trial participants only receive assigned intervention	
▪ Rationale and techniques for reducing contamination discussed at monthly site meetings with health facility and trial staff	
▪ Information regarding participant’s trial arm allocation is kept in a locked drawer that can only be accessed by RA	
▪ Contamination is measured quarterly as a component of checklist to measure the degree of fidelity [63]	
*Strategies to ensure treatment fidelity*	
▪ Fidelity checklists completed by RA (self-administered) and observer. Key issues discussed at quarterly staff meetings	
▪ Intervention sessions audio recorded, and 10% reviewed centrally	
▪ Electronic time-stamps of session start and end time and bi-monthly supervisions by trial coordinator	
▪ Unannounced trial coordinator visits	
▪ Plans for implementation setbacks which include two providers (RAs) per site, back-up tablets, paper forms, power banks, and back-up physical space for intervention implementation	
Analysis	
▪ If contamination occurs, we will perform contamination-adjusted intention-to-treat analysis [64]	
▪ The degree, nature, and effects of contamination will be included in final manuscripts	

### Participant follow-up surveys

After enrollment, participants will attend trial visits at months 1, 3, 6, and 12 for a total of five trial visits. Participants will be provided with the equivalent of $5 USD for transport reimbursement for each trial visit. Follow-up surveys will be completed at a time and place separate from routine ART refill visits.

### Measures

A full list of measures and a schedule of assessments are outlined in Table 3. See also the Standard Protocol Items: Recommendations for Interventional Trials (SPIRIT) checklist, provided as Additional file 1. The primary outcome is a composite measure of retention and viral suppression at 6 and 12 months. Participants will be considered retained in care if they are verified by clinic records as active on ART within 2 months of the last trial visit. Viral suppression will be measured as viral load < 1000 copies/mL. Retention was chosen as a primary outcome due to its strong association with adherence [81]. Viral suppression as a marker of adherence was included in the primary outcome because viral suppression is the clinical goal of ART and leads to decreased HIV-related mortality and morbidity [82].

**Table 3:** VITAL Start trial outcomes and schedule of measures

Schedule (month number)	0	1	3	6	12
Informed consent	x				
Allocation	x				
Intervention	x				
Aim 1: Retention and adherence (viral load [VL] < 1000 copies/ml) at 12 months					
Retention in care (data abstraction from facility records)		x	x	x	x
Viral suppression (VL < 1000 copies/ml)				x	x
*Aim 1b: Behavioral adherence*					
Self-reported adherence [66, 67]		x	x	x	x
ART side effects		x	x	x	x
Pharmacy refill data abstraction [68]		x	x	x	x
Tenofovir diphosphate level					x
*Aim 1c: Knowledge and psychosocial impact*					
HIV/ART knowledge and attitudes survey [69]	x		x		x
Adherence self-efficacy [70, 71]	x	x		x	x
Motivation and behavior skills assessment: LifeWindows tool [72]		x		x	x
Self-reported partner disclosure and World Health Organization intimate partner violence survey [73]	x	x		x	x
Multidimensional Scale of Perceived Social Support (MSPSS) [74] and self-reporting questionnaire [75]	x	x		x	x
Shortened Alcohol Use Disorders Identification Test [76] and Drug Use Disorders Identification Test [77]	x	x		x	x
Patient-Provider Relationship Scale [78, 79]		x			x
Aim 2: Implementation outcomes					
Participant satisfaction surveys	x				
Interviews with participants, partners, health care workers	x	x	x	x	x
Trial fidelity evaluations	x				
Contamination assessment		x		x	x
Time-motion assessments in line with Suggested Time And Motion Procedures (STAMP) checklist [80]	x				
Aim 3: Cost-effectiveness analysis. Data collected throughout trial period. Start-up: video creation, training, tablets and accessories (security cable, power bank). Recurring: tablet maintenance; personnel time and salaries					

We will also examine several secondary outcomes. Adherence will be further measured by self-reported adherence, pharmacy refill data, and tenofovir diphosphate concentration at 12 months. Tenofovir diphosphate (TFV-DP) is a metabolite of tenofovir that allows estimation of average adherence over the preceding 2 weeks. Use of TFV-DP will allow detection of small, clinically significant differences in adherence between groups that may be missed by viral load testing alone [83].

To critically examine the implementation of VITAL Start, we will also examine participant satisfaction, intervention fidelity, contamination, and time-motion data. Guided by social action theory [84], we will also assess a series of behavioral, mental health, and psychosocial measures during the trial as potential mediators or modifiers of the intervention’s effect as well as potential independent factors, which may influence adherence behaviors. These include assessments of HIV knowledge, whether a patient is a new ART initiator or re-initiating ART, measures of depression, alcohol and substance use, intimate partner violence, and availability of social support. Finally, we will collect financial data alongside clinical outcomes to assess the cost-effectiveness of VITAL Start compared to SOC.

The issue of distinguishing trial retention from retention in clinical care deserves special mention in this trial, where retention in clinical services is a primary outcome, and efforts to promote trial retention may have unintended consequences. Trial staff will collect telephone numbers and/or physical address details from participants willing to provide this information. When a participant is absent for a trial visit, staff will trace her first by telephone, and will make three valid attempts (defined as speaking with the participant) or will attempt daily for a week, whichever comes first. After phone tracing, staff will attempt to visit the participant at home, and will make up to three valid attempts. Participants will be allowed to complete trial questionnaires at home during these tracing attempts if they desire and a secure location is available for the interaction. All tracing attempts will be recorded to allow analysis of tracing attempts according to trial arm and controlling for tracing attempts, if necessary.

Participants who transfer to a different facility will not be terminated from the trial unless they withdraw consent or are found to have died. This decision reflects the highly mobile nature of the trial population that we have observed in previous studies. By keeping participants enrolled, they can easily transition back into the trial if they move back to the catchment area or are willing to continue attending trial visits. If a subject is discontinued at any time after entering the trial, the trial team will try and ensure that the subject completes the Final Visit survey and procedures.

### Sample size

The sample size calculation is based on a comparison of the proportions of those in the intervention versus SOC arms who reach the combined primary endpoint at 12 months after maternal ART initiation. A combination of various scenarios was considered for retention in care (50–90%), maternal viral suppression (50–90%) among those retained, and contamination rate (0–15%). A difference of 15% was selected as the smallest difference that would be of clinical significance.

For the primary analysis in all subjects, accounting for up to a 15% contamination (pilot data suggested 0%) and 5% attrition rate (based on transfer-out rates from MOH data at trial clinics in 2016) [3], and assuming a 1:1 allocation ratio to achieve 90% power to detect a minimum difference of 15% between the control and intervention groups, 796 subjects are required (398/398 per arm) at a significance level of 0.025 to account for multiple comparisons. We further increased this by 12% to 892 total (446 per arm) to account for a potential change to national guidelines to recommend dolutegravir-based regimens as first-line ART. The higher potency of dolutegravir-based regimens may allow higher rates of viral suppression at lower adherence levels, therefore improving overall viral suppression and making a difference between arms more difficult to detect.

### Assignment of interventions and blinding

Participants will be randomly assigned in a 1:1 fashion stratified by clinic proportional to the number of potential eligible participants at that site. Smith’s randomization algorithm will be applied for random assignment to VITAL Start or SOC to reduce imbalance throughout the randomization process [85]. The primary trial biostatistician will generate serial random codes for each clinic and place them on a sequential list that will be maintained by the trial coordinator. Assignments will be provided to RAs in opaque sealed numbered envelopes to be opened sequentially prior to administration of the baseline survey. If a participant refuses to participate in the trial after randomization, the next subject will not replace her. The randomized participant who refused participation will be included in the intention-to-treat (ITT) analysis. Due to the nature of the intervention, RAs, clinic staff, and participants will not be blinded. Separate research staff (outcome assessors) will remain blinded and conduct the outcome assessments including follow-up surveys. They will not be informed of the participants’ group assignment. All trial investigators will also remain blinded. After data collection is complete and the database cleaned and locked, a research team member will break the randomization code to input the group allocation within the pre-existing data set and enable between-group analyses.

Strategies for ensuring fidelity to the VITAL Start intervention are described in detail in Table 2. Fidelity will be quantitatively assessed by both the RA performing the counseling and an RA observing the counseling session. Contamination may occur if participants who receive the VITAL Start intervention discuss elements of the video with participants who received standard of care (SOC). Pilot data suggested little to no risk of contamination with the contamination reduction procedures that will be used in this trial (Table 2). However, in case contamination does occur, we will assess the prevalence, magnitude, and source of contamination. Participants will complete a contamination assessment at months 1, 6, and 12, and we will account for contamination via a contamination-adjusted ITT analysis [64].

### Data collection and management

All data will be collected on electronic case report forms (CRFs) by trained trial staff using electronic CRFs on tablets and will be monitored weekly via data checks by the study team. We elected to use electronic CRFs to improve both efficiency and data quality through the use of internal checks and automated skip patterns [86]. Backup paper CRFs will be maintained at all sites. The use of tablets also allows us to use an audio computer-assisted self-interview approach to assess self-reported adherence to medication, theoretically improving participants’ comfort with responding truthfully to these questions [87]. RAs will administer surveys at baseline; all follow-up surveys will be conducted by blinded outcome assessors at a location separate from the ART clinic where they receive their ART medications.

All tablets will be encrypted and password-protected and will use a secure connection to transfer data to a central server on a daily basis. A full list of data sources and schedule of assessments can be found in Table 3.

### Statistical methods

The primary analysis will be conducted as ITT. If there are exceptional circumstances which could lead to excluding randomized subjects from the ITT population without the possibility of introducing bias (e.g., an ineligible patient is mistakenly enrolled), a modified ITT (mITT) analysis will be used as the primary analysis. A statistical analysis will be performed at the end of the trial; no statistical interim analysis is planned.

Baseline characteristics by arm will be displayed using descriptive statistics. The primary outcome measure (maternal ART retention and viral suppression) is a binary variable, and it will be compared by chi-square tests at 12 months, and followed by logistic regression to study the association between intervention and outcome while controlling the baseline prognostic factors of the main outcome. The main exposure variable and any factor deemed to be clinically relevant will be forced into the model regardless of the statistical significance. Secondary outcomes include components of the composite outcome, self-reported adherence, pharmacy refill data, ART knowledge and self-efficacy, motivation and behavior skills, partner disclosure, and social support, etc. For the 12 months outcome, chi-square tests and two-sample *t* tests will be used. For repeated measures, the generalized estimating equations (GEE) method and linear mixed models will be used to examine differences in trajectories over time between the intervention and control groups while accounting for the correlation among repeated measures from the same subject. Additionally, for time-to-event outcomes, such as time to partner disclosure, we will conduct survival analysis using the Kaplan-Meier (KM) method with log-rank test and Cox proportional hazard models including the covariates. A *p* value of 0.05 will be deemed statistically significant and a *p* value of 0.1 regarded as a statistical trend. All analyses will be performed using SAS software (SAS Institute, Inc., Cary, NC, USA).

### Cost-effectiveness analysis

The MOH will be the main implementer of the VITAL Start intervention as well as SOC pre-ART counseling. Therefore, we will assess costs from the provider (MOH) perspective. We will use both micro- and macro-based approaches as appropriate to identify and quantify intervention resources [88–90]. Retrospectively, we will extract information on resources expended for video design and production from inventory and other program documents. Unit prices will be obtained from account records or from local retailers. To measure staff time devoted to pre- ART counseling, we will perform time-motion assessments in line with the Suggested Time And Motion Procedures (STAMP) [80, 91, 92]. We will sample sufficiently to account for potential sources of heterogeneity such as trial clinic and provider. The value of staff time will then be estimated as the product of their gross salary and share of time allocated to intervention/pre-ART counseling.

We will calculate the incremental cost-effectiveness ratio (ICER) for the primary outcome of economic interest. The ICER is the difference in costs between two interventions divided by the difference in their effects, and it can be interpreted as the incremental price of a unit health effect from the intervention under study, as compared to the alternative. We will conduct one-way and multiway sensitivity analyses to model cost-effectiveness under various conditions, such as lower pricing of video production, mode of delivery, tablets, and alternate health care worker (HCW) cadres to deliver the intervention. Finally, we will conduct probabilistic sensitivity analyses using bootstrapping methods to ascertain the robustness of our results to extreme assumptions/scenarios. Based on emerging literature [89, 93], we will determine cost-effectiveness by comparisons with published ICERs for other commonly implemented HIV interventions in Malawi and southern Africa, as well as with gross domestic product (GDP)-based thresholds (0.5× and 1× the Malawi per capita GDP of $487) [94].

### Monitoring

Blinded data monitoring will be conducted using descriptive statistics for quality assessment purposes, such as monitoring subject accrual and compliance as well as identifying outstanding values, etc., for data quality control. The results will be displayed for follow-up measures for the combined group, and otherwise by blinded treatment groups. We will monitor for overall trends in retention weekly. All unanticipated problems and adverse events will be recorded on designated CRFs and reported to the principal investigator (PI) and appropriate regulatory bodies. All potential adverse events will be reviewed weekly by the PI, trial coordinator, and research supervisors. The data manager will review trial data weekly. A stakeholder advisory group (SAG) comprising trial site supervisors, trial site clinical staff, and community members will meet twice a year to provide input on study implementation. In addition, an external monitor will evaluate trial operations annually.

Measures to ensure intervention implementation and fidelity are detailed in Table 2.

### Ethics and dissemination

This trial has been approved by the Baylor College of Medicine Institutional Review Board (IRB; protocol H-39785) and the Malawi National Health Sciences Research Committee (NHSRC; protocol 16/05/1593). If the investigators elect to make important modifications to the protocol, amendments will be submitted to both the Baylor IRB and Malawi NHSRC.

Informed consent will be obtained in writing from all participants by trial site staff using forms in Chichewa approved by both the Baylor IRB and Malawi NHSRC. A staff member fluent in Chichewa will be responsible for reviewing the form in detail and eliciting any questions before the participant signs. If the patient is unable to read, staff will read the consent form to the participant in its entirety, the participant will mark her thumbprint to indicate consent, and the process must be witnessed by a third party not involved in the trial.

Confidentiality will be prioritized and will include the following measures specific to our intervention and trial population:

1. We will use only study identification numbers for all data collection and analysis.
2. All electronic data will be secured through encryption, and paper data will be secured through locked cabinets stored in locked study offices.
3. We will ensure that all staff are trained to the highest standards of confidentiality and data security procedures through appropriate online modules provided by the Collaborative Institutional Training Initiative (CITI).
4. When conducting home visits, staff will not wear anything that shows they are from a health facility or the trial, and they will not carry information identifying the participant as having HIV, to minimize unintentional risk of disclosure.

### Ancillary and post-trial care

All participants will receive free lifelong ART as per Malawi MOH guidelines. Lab results from the trial (viral load measurements and TFV-DP concentration) will be provided to participants after the final trial visit in writing, along with a brief interpretation of the results for the participant’s clinician.

We will ensure that trained psychosocial counselors will be available to support participants who screen positive for current intimate partner violence, depression, or suicidality.

### Dissemination policy

Results will be reported to the MOH and local stakeholders, as well as at local and international conferences. Results will also be submitted to peer-reviewed journals. Authorship eligibility will be guided by the International Committee of Medical Journal Editors criteria [95].

## Discussion

By evaluating a brief, low-cost, scalable and locally developed and culturally tailored tool to promote adherence and retention, this trial has the potential to streamline ART counseling in resource-limited settings. In contrast to many other interventions, it also has the potential to liberate precious HCW time, thereby supporting health systems in meeting the needs of their patients.

This trial utilizes multiple methods to examine adherence, including two methods of behavioral adherence (self-report and pharmacy pill count), as well as viral load and TFV-DP testing. Due to TFV-DP’s longer half-life, its concentration is a measure of average adherence over the previous 2 months, allowing estimation of the average number of doses missed per week. Use of this marker thereby allows reliable detection of clinically significant between-group differences in adherence. This trial also utilizes a combined outcome of retention and viral suppression. Further, the trial not only seeks to evaluate effectiveness, but it also aims to critically examine the implementation of a new intervention through qualitative interviews, validated questionnaires, time-motion measures, and cost-effectiveness analysis, among other methods.

As with any research design, the trial has potential limitations. In studies where a primary outcome is retention, caution is warranted in determining how participants are traced for missed trial visits, since the act of missed trial visit tracing plausibly affects clinical retention. We attempted to address this concern by outlining precisely how many tracing attempts for missed trial visits should be done, and we developed a mechanism for recording all missed trial visit tracing attempts to allow incorporation of this effect into the final analytic model as necessary.

A small pilot study suggested the VITAL Start intervention to be an acceptable, feasible approach that resulted in excellent knowledge acquisition and better short-term self-reported adherence as compared to the control. This multisite randomized controlled trial will allow rigorous evaluation of this promising approach to improve ART adherence.

### Trial status

The protocol is version 4.0 dated 23 August 2018. Trial enrollment started in October 2018, and recruitment will be completed in March 2021.

## Supplementary information

**Additional file 1.** SPIRIT 2013 checklist: recommended items to address in a clinical trial protocol and related documents.

## Data Availability

Data sets generated and/or analyzed during the current trial are available from Baylor-Malawi on reasonable request.

## Abbreviations

AIDS: Acquired immune deficiency syndrome
ANC: Antenatal care
ART: Antiretroviral therapy
AUDIT: Alcohol Use Disorders Identification Test
B +: Option B+
CITI: Collaborative Institutional Training Initiative
CRF: Case report form
DUDIT: Drug Use Disorders Identification Test
GDP: Gross domestic product
GEE: Generalized estimating equation(s)
HCW: Health care worker
HIV: Human immunodeficiency virus
ICER: Incremental cost-effectiveness ratio
IMB: Information-motivation-behavioral
IRB: Institutional Review Board
ITT: Intention to treat
KM: Kaplan-Meier
mITT: Modified intention to treat
MOH: Ministry of Health
MSPSS: Multidimensional Scale of Perceived Social Support
NHSRC: National Health Sciences Research Committee
NIH: National Institutes of Health
PMTCT: Prevention of mother-to-child transmission
Q&A: Question and answer
RA: Research assistant
SD: Standard deviation
SMS: Short message service
SOC: Standard of care
SRQ: Self-reporting questionnaire
STAMP: Suggested Time And Motion Procedures
STD: Sexually transmitted disease
TFV: Tenofovir
TFV-DP: Tenofovir diphosphate
USD: United States dollar
VITAL: Video intervention to Inspire Treatment Adherence for Life
VL: Viral load
